# Picomolar or beyond Limit of Detection Using Molecularly Imprinted Polymer-Based Electrochemical Sensors: A Review

**DOI:** 10.3390/bios12121107

**Published:** 2022-12-01

**Authors:** Naheed Sidiq Shah, Vandana Thotathil, Shabi Abbas Zaidi, Hanan Sheikh, Maimoona Mohamed, Ahmadyar Qureshi, Kishor Kumar Sadasivuni

**Affiliations:** 1 Department of Chemistry and Earth Sciences, College of Arts and Sciences, Qatar University, Doha P.O. Box 2713, Qatar; 2 Center of Advanced Materials, Qatar University, Doha P.O. Box 2713, Qatar

**Keywords:** molecular imprinted polymer, limit of detection, ultrasensitive, electrochemical sensors

## Abstract

Over the last decades, molecularly imprinted polymers (MIPs) have emerged as selective synthetic receptors that have a selective binding site for specific analytes/target molecules. MIPs are synthetic analogues to the natural biological antigen–antibody system. Owing to the advantages they exhibit, such as high stability, simple synthetic procedure, and cost-effectiveness, MIPs have been widely used as receptors/sensors for the detection and monitoring of a variety of analytes. Moreover, integrating electrochemical sensors with MIPs offers a promising approach and demonstrates greater potential over traditional MIPs. In this review, we have compiled the methods and techniques for the production of MIP-based electrochemical sensors along with the applications of reported MIP sensors for a variety of analytes. A comprehensive in-depth analysis of recent trends reported on picomolar (pM/10^−12^ M)) and beyond picomolar concentration LOD (≥pM) achieved using MIPs sensors is reported. Finally, we discuss the challenges faced and put forward future perspectives along with our conclusion.

## 1. Introduction

**Figure 1:**
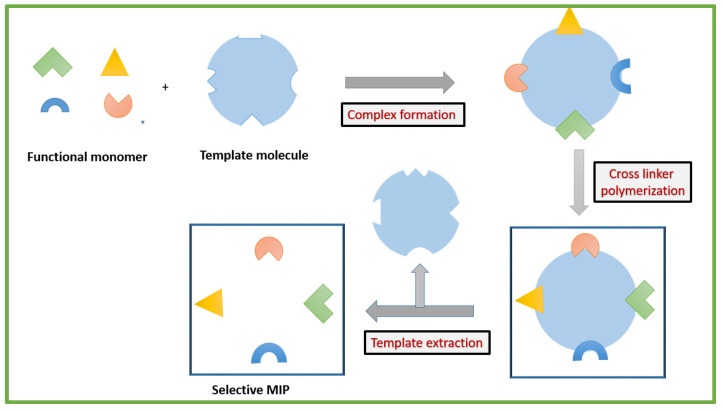
Scheme depicting the stages of preparation of MIPs.

Molecularly imprinted polymers (MIPs) are polymeric matrices obtained using molecular imprinting technology (MIT) [1,2,3]. This is a versatile synthetic technique used to design artificial receptors with selective sensing for a specific analyte [4,5,6]. MIPs can mimic natural biological antibody–antigen receptors and operate using a “lock and key” mechanism for selective and specific binding to the imprinted molecule. The general synthesis of MIPs follows the same basic procedural steps sequentially, which include the following: (1) polymerization of functional monomers and cross-linkers in the presence of the target molecule and a suitable porogenic solvent. In the electro-synthesis of MIPs, the template is electro-polymerized in the presence of a suitable monomer via potential application. (2) polymerization is followed by removal of template molecules. This process is facilitated by solvent extraction, chemical decomposition, or potential application in order to break the bonds formed between template and monomer. Removal of the template leaves behind a target specific cavity available for rebinding. (3) Finally, MIPs are exposed to a target containing sample where the cavity selectively uptakes the complementary target molecules [7,8,9,10,11,12,13]. A schematic representation of general MIP preparation is shown in Figure 1.

Research in the field of MIPs has received considerable attention due to the advantages it exhibits i.e., high level of specificity, stability and binding affinity for the target analyte, stability under different environmental conditions, and its low cost [14,15,16,17]. Furthermore, these synthetic biomimetic polymeric matrices are quite stable and may be kept for a long time without requiring special storage conditions, which is very important in terms of saving money, time, and effort [18,19]. Thus, MIP is considered an excellent technique for producing any target molecule, especially small molecules [20]. MIPs have been employed in a variety of applications where selective binding events are important, such as biosensors, nanocarriers, proteins (macromolecules), drug delivery systems, diagnostic and imaging, immunoassays, affinity separation, directed synthesis and catalysis [21,22,23,24,25,26,27]. MIP techniques are also very important in the biomedical field because they can mimic physiologic recognition units in the body, such as receptors and antibodies [28,29].

The limit of detection (LOD) is defined as the lowest concentration of an analyte that can be consistently detected with an appropriate material. Achieving high LOD or ultra-low abundance of an analyte of interest is beneficial in diverse areas such as water, food, soil, and biological samples. For example, this is particularly necessary to analyze real-life samples such as urine, serum, or blood for various disease markers for early diagnosis. Hence, synthesizing materials to achieve high LOD is one of the critical parameters in various applications. Generally, MIPs offer a highly sensitive response and the lowest possible limit of detection (LOD) in a short time because of their selective features. The electrical signal produced by electrochemical-imprinted polymer-based sensors is proportional to the concentration of the target analyte. Furthermore, to develop MIPs, selecting the proper polymeric material and imprinting process remarkably affects the efficiency of the process and hence its capacity for a low detection limit. The developed reusable electrochemical imprinted sensor shows promising applications in the health sector for the detection of specific proteins in lower detection limits.

**Figure 2:**
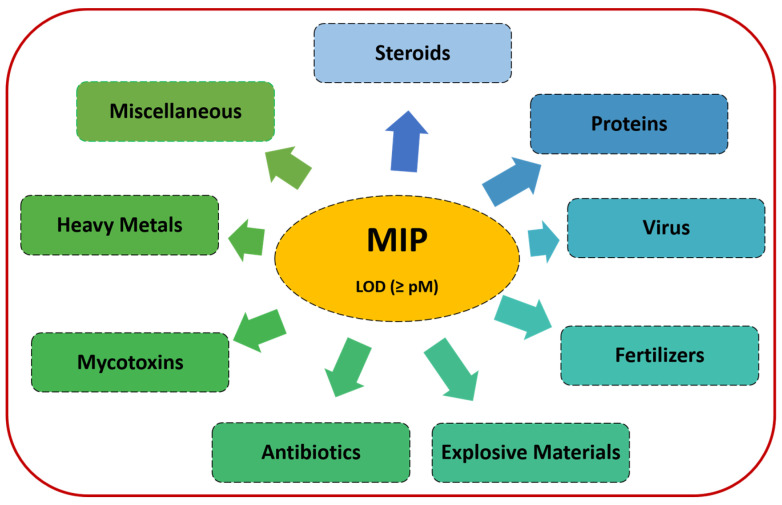
Summary of target analytes discussed in the review.

Thus, this review works focuses on the picomolar (pM or 10^−12^ M) and beyond concentration LOD achieved via MIP sensors. Herein, we comprehensively discuss the approaches listed for various sensor probe designs and their sensing strategies that paved the way for the detection of picomolar concentrations of analytes. For convenience, this review has been categorized based on the types of target analytes as shown in the schematic in Figure 2. We also highlighted future prospects of MIPs for the achievement of ultrasensitive LODs.

## 2. General Synthesis Procedure of an MIP

In molecularly imprinted polymer (MIP) synthesis, a target analyte (template) is functionalized with a monomer followed by cross-linking polymerization under certain experimental parameters to obtain a 3D polymer matrix. Several polymerization methods are employed for the synthesis of MIPs, such as precipitation, bulk, sol-gel, suspension, emulsion, and electropolymerization. For the synthesis of electrochemical MIP sensors and biosensors, electropolymerization is frequently used [30,31,32].

Generally, covalent and non-covalent approaches are used where covalent and non-covalent bonds form between the template and monomer, respectively. Later, the template extraction (or removal) is carried out with the help of mild solvents or a mixture of an organic solvent and acid, or voltage application when the bonds formed between the template and monomers are broken, leaving behind cavities complementary to the shape and size of the template. Despite the high fidelity and specificity of cavities created via the covalent approach, the non-covalent approach is prevalent as the non-covalent bonds are easily broken under mild experimental conditions with almost no distortion in the shape of the imprinted cavities compared to covalent bonds that require harsh conditions [33,34,35]. We recommend our readers to read some excellent works published on MIPs in the last decade [36,37,38,39].

### Polymerization Method of Molecular Imprinting

Brief descriptions of the various polymerization methods involved in the synthesis of MIPs with merits and demerits are given below [40,41,42].

Bulk Polymerization: This is one of the most prominent and conventional methods wherein the template, monomer, porogenic solvent, initiator, and crosslinker are combined in the same container followed by polymerization initiation, either thermally or via UV radiation. The resulting bulk polymer thus obtained has to be further crushed, ground into a small size, and sieved. Merits—Ease of fabrication, less contamination, no additional solvent required, and low cost. Demerits—Irregular particle size, destruction of interaction site during grinding, the possibility of sample overheating, time consuming and labor intensive.

Suspension Polymerization: In this method, a pre-polymer mixture is made by dissolving the template molecule, monomer, initiator, and crosslinker. Further, the droplets of the formed mixture are suspended in a dispersion medium. A stabilizing medium is used to stabilize the mixture in dispersion medium, which results in the formation of spherical beads of sizes in the range of µM to mM. Some of the commonly used stabilizers are poly(vinyl alcohol), poly(vinyl acetate) poly(vinyl pyrrolidone), and cellulose acetate butyrate. Merits—MIP film with high porosity, as well as a regular size and shape. Demerits—Requirement for a reaction mixture with aqueous and organic phases, stabilizer and initiators are mandatory, initiator and monomer are hydrophobic, poor recognition and large particle size.

Precipitation Polymerization: High-quality monodisperse MIP microspheres are produced in a large excess of porogenic solvent without the use of stabilizers and emulsifiers. Microspheres are produced by entropic precipitation of the primary molecules followed by the capture of oligomers from the solution. Since the growing polymer chain has a higher density than the solvent, the polymer precipitates out of the solution. Merits—Simple procedure consuming less time; uniformity in size; stabilizer and emulsifier are not required. Demerits—polymerization is achieved when the density of the polymeric chains and the dilution factor are high.

Multistep swelling Polymerization: Uniformly sized seed particles are dissolved in water followed by the addition of organic solvents. When the initial particles swell to the expected size, all the required components are added and incorporated, and then polymerization is induced. Merits—Monodispersed beads of controlled diameter can be obtained. Demerits—Reaction conditions, and procedures are complicated and require aqueous emulsions 

Surface Polymerization: In this method, surface grafting of the MIP layer is performed using several techniques to restrain the surface polymerization at the surface of the beads. A very thin imprinted surface layer is obtained, which is selective for specific molecules.Merits—Monodispersed product obtained. Demerits—Time consuming and complicated.

In situ Polymerization: This method combines the advantages of molecularly imprinted technology and monolith column. The advantages include simplicity and the possibility of carrying out the process in situ, in a single step using a free-radical molding process directly on a chromatographic column or transducer. Merits—The density and thickness of the MIP layer can be controlled. Homogenous coating. Fast response to the template. Demerits—Difficult to remove the template. Slow kinetics of removal and rebinding.

Electro-polymerization: In this type of polymerization, the template is mixed with a suitable monomer, and potential is applied for the polymerization. Merits—Fast polymerization; the film thickness is controllable, allowing a superior adhesion of the polymer to the transducer surface. Demerits—the polymer film’s short lifespan, easy fouling due to the formation of redox product.

## 3. Determination of Various Analytes via MIPs

For convenience, we have categorized the various analytes (imprinted molecules) in their most suitable form. In the following section, each of the MIPs will be discussed briefly.

Table 1 summarizes the various analytes depicting linear ranges, LOD/LOQ*, detection methods, and% recoveries in real samples using different MIP matrices.

### 3.1. Steroids, Hormones, and Metabolites

Cholesterol (CHO) is a steroidal metabolite and an essential lipid for the human body. For an adult, the optimum plasma cholesterol should be less than 5.2 mM and anything higher than 6.2 mM would be considered a health threat, which might lead to coronary heart disease or myocardial and cerebral infarction. For the development of a CHO imprinted sensor by Jian et al., CHO was electropolymerized with poly-amino thiophenol (p-ATP) as a functional monomer over gold nanoparticles (AuNPs) functionalized multiwalled carbon nanotubes (MWCNTs) over glassy carbon electrode (GCE). The characterization of the imprinted CHO sensor by using cyclic voltammetry (CV), differential pulse voltammetry (DPV), and scanning electron microscopy (SEM) studies indicated that CHO molecules attached through hydrogen bonding on the surface of AuNPs–MWCNTs/GCE, thereby increasing the imprinted site, resulting in an LOD of 0.33 pM [43]. Classified as an endocrine active compound, 17-β-estradiol is a steroid hormone responsible for the regulation of the female reproductive system. Its rapid, selective, and sensitive detection is intensively required. Florea et al. reported the detection of 17-β-estradiol by electropolymerizing it with p-ATP functionalized AuNPs. Extraction of the resulting template enabled the forming of cavities with high affinity and recognition for binding estradiol. The performance of the developed MIP-Au sensor was evaluated by incorporating hexacyanoferrate/hexacyanoferrite as a redox probe and monitored via linear sweep voltammetry (LSV) [44]. In another study, Liu and colleagues fabricated a 17-β-estradiol imprinted polymer-based impedimetric sensor in the presence of a newly synthesized 3,6-diamino-9-ethyl carbazole monomer (modified carbazole monomer) via 25 cyclic voltammetry (CV) scans over GCE. The SEM and transmission electron microscopy (TEM) results indicated the formation of islands like rough and rugged microstructures with an average diameter and height of 25.47 nm and 20.26 nm, respectively. The optimized monomer/template of 10 was found to be most suitable for the synthesis of MIPs with the highest sensitivity. After optimal conditions, the sensor demonstrated a large linear range, low LOD and high affinity, and selectivity towards structural analogs. The analytical response of the developed MIPs indicated that the modified carbazole monomer possesses active selective 17-β-estradiol binding sites and high-charge carrier mobility [45]. 

**Figure 3:**
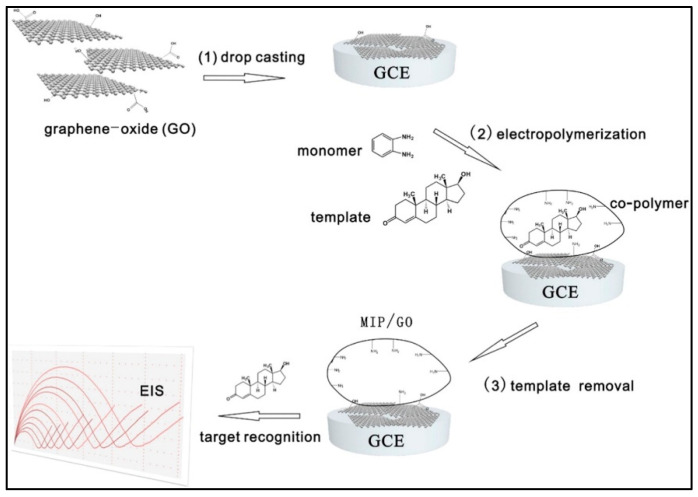
The process for the preparation of the MIP/GO composite. (1) Drop-casting graphene-oxide sheets on the GCE surface; (2) Electropolymerization of the MIP layer on the surface of GO modified electrode; (3) Template removal and recognition of the target testosterone in different concentrations ([47]).

Testosterone is a primary androgen hormone with a significant effect on human health, detection of which is important in both clinical analysis and in the sporting industry to prevent doping. Hence, for rapid, sensitive detection of testosterone in urine, a novel water-soluble macroporous molecularly imprinted film (MIF)-based surface plasmon resonance (SPR) sensor was developed. The process of synthesis of MIF involved photo copolymerization of monomers methacrylic acid (MAA) and 2-hydroxyethyl methacrylate [HEMA]) using a cross-linker (ethylene glycol dimethacrylate, EDMA), and polystyrene nanoparticles (PSNPs) in combination with template testosterone molecules. Subsequent removal of template molecules along with polystyrene nanoparticles (PSNPs) resulted in the macroporous structure of MIF with specific recognition sites for testosterone. Under optimized experimental conditions, the proposed sensor exhibited femtomolar range LOD toward testosterone in PBS and artificial urine (AU) in the presence of other structurally related molecules, including progesterone and estradiol, with an extended shelf-life of over 8 months. Despite the comparatively high cross-selectivity of estradiol, this SPR sensor exhibited the lowest LOD reported ever [46]. In another study reported for testosterone, Liu et al. developed an ultrasensitive Electrochemical Impedance spectroscopy (EIS, impedimetric) sensor by electropolymerization of testosterone with o-phenylenediamine (*o*-PD) by 30 cycles of CV scans in graphene-oxide (GO) sheets cast over GCE, as shown in Figure 3. The optimized monomer/template of 20 was a deciding factor in the achievement of high binding affinity sites. The alterations in non-faradaic interfacial impedance due to the structure and large surface area of the GO nanosheet validated the low detection limits under optimal conditions [47].

Follicle-stimulating hormone (FSH) is one of the gonadotropic hormones produced by the pituitary gland. Determining levels of FSH helps in the identification or treatment of reproductive disorders. Monitoring FSH levels in polycystic ovarian syndrome (PCOS), an endocrine disorder, is vital. A novel nano-molecular highly selective FSH-MIP sensor was developed using electro polymerization of MAA and EDMA on a NiCo_2_O_4_/rGO nanocomposite-modified indium tin oxide (ITO). Contact angle measurements were performed to confirm the complete template removal and surface energy. Pareek et al. reported an effective and sensitive sensor for the electrochemical detection of FSH in PCOS [48]. Somatostatin (SOM) is considered a universal endocrine molecule and a peptide hormone in the central nervous system (CNS). Highly selective and sensitive recognition of SOM was achieved by developing a porous MIP by Ozkan et al. The SOM was photopolymerized in the presence of functional monomer, N-methacryloyl-L-aspartic acid (MAAsp) and HEMA, matrix monomer, and EGDMA as a cross-linker on a GCE to obtain a P(HEMA-MAAsp)@MIP/GCE sensor. Characterization techniques such as SEM, Fourier-Transform Infrared spectroscopy (FTIR), and EIS were utilized for examining the P(HEMA-MAAsp)@MIP/GCE sensor. Under ideal conditions, the P(HEMA-MAAsp)@MIP/GCE sensor offered a femtomolar detection limit and limit of quantification (LOQ) of 0.584 fM [49].

### 3.2. Proteins

**Figure 4:**
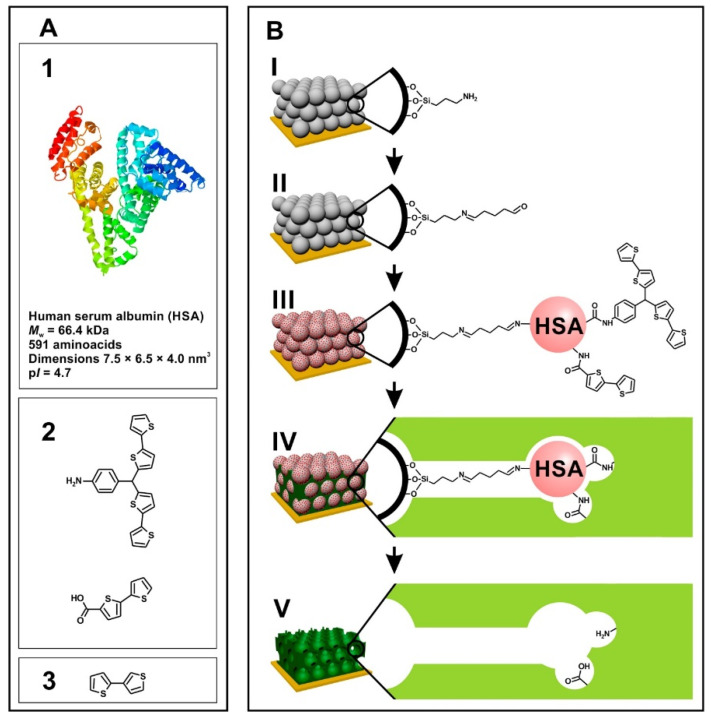
(**A1**) 3-D structure of HSA (http://www.rcsb.org/pdb/explore/explore.do?pdbId=1E7H (accessed on 11 October 2022), structural formulas of (**A2**) functional monomers; (**A3**) the cross-linking monomer. (**B**) Illustration of the elaboration procedure of the poly(2,3′-bithiophene) inverse opal imprinting with HSA ([50]).

Human serum albumin (HSA) is the most abundant protein in human plasma. To determine the level of HSA, hierarchical nanostructured artificial receptor materials were prepared. The production of MIP film in the shape of an inverted opal was achieved due to colloidal crystal templating by using silica NPs and improved electrochemical polymerization of 2,3′-bithiophene via semi-covalent imprinting. The semi-covalent approach produced geometrically homogenous and well-defined imprinted cavities. Figure 4 illustrates the preparation scheme of the opal imprinting method for HSA.

For the imprinting, propylene carbonate (PC) was used as a solvent in which HSA was insoluble, thus preventing the extraction of water from HSA and leading to highly specific three-dimensional cavities after the removal of HSA under strongly alkaline conditions. This provided an improved specific surface area of imprinted cavities existing within the pores. Furthermore, due to earlier derivatization of HSA molecules with suitable functional monomers, all binding sites were positioned on the surface of the imprinted cavities at places matching the positions of functional groups present on the surface of HSA molecules. These three levels of improvement and the integration of MIP with an extended gate field effect transistor (EGFET), were able to detect HSA in the femtomolar concentration range with a sensitivity of 118 µA/µM [50]. Myoglobin (Myo), an iron- and oxygen-binding protein, is found in cardiac and skeletal muscles. For early detection of myocardial infarction (MI), monitoring Myo, an important early-onset biomarker, is essential. A fluorescent biosensor based on cadmium telluride (CdTe) quantum dots (QDs) was constructed with mercaptopropionic acid (MPA). Plastic antibodies on the exterior surface of aqueous soluble QDs were tailored to produce a highly sensitive fluorescent detection probe. Furthermore, the target protein was incubated with QDs and further modified via radical polymerization of acrylamide and bisacrylamide to achieve surface imprinting. Conjugation of plastic antibodies with QDs enabled detection up to 7.6 fM, which was achieved due to the high quenching abilities of specific binding of Myo on imprinted cavities, regardless of preparation methods of MIPs and NIPs (i.e., bulk and surface imprinting) and incubation time. The adsorption kinetic study showed that most of the imprinted cavities were saturated within 30 min of incubation (1.5 nM Myo) as displayed by a rapid decrease in the fluorescence intensity. Then, the thermodynamic equilibrium was reached. Moreover, as expected, the surface-imprinted MIPs outperformed the bulk-imprinted MIPs [51]. Amyloid-β oligomer (AβO) accumulation is believed to be the most relevant indicator for the progression of Alzheimer’s disease (AD), the most common form of dementia globally. For improved diagnosis of AD, highly selective and sensitive detection of the potential biomarker AβO was developed using MIP with aptamer as a target receptor. The aβO-specific aptamer was immobilized on the surface of silver and silica nanoparticles (SiO_2_@Ag NPs). MIP/target/aptamer sandwich assay was able to generate a highly selective electrochemical signal by utilizing a small amount of AβO to activate many electrochemically active AgNPs. The developed biosensor was able to determine up to 1.22 pM of AβO, so is suitable for early detection of AβO in dementia patients [52].

### 3.3. Virus

**Figure 5:**
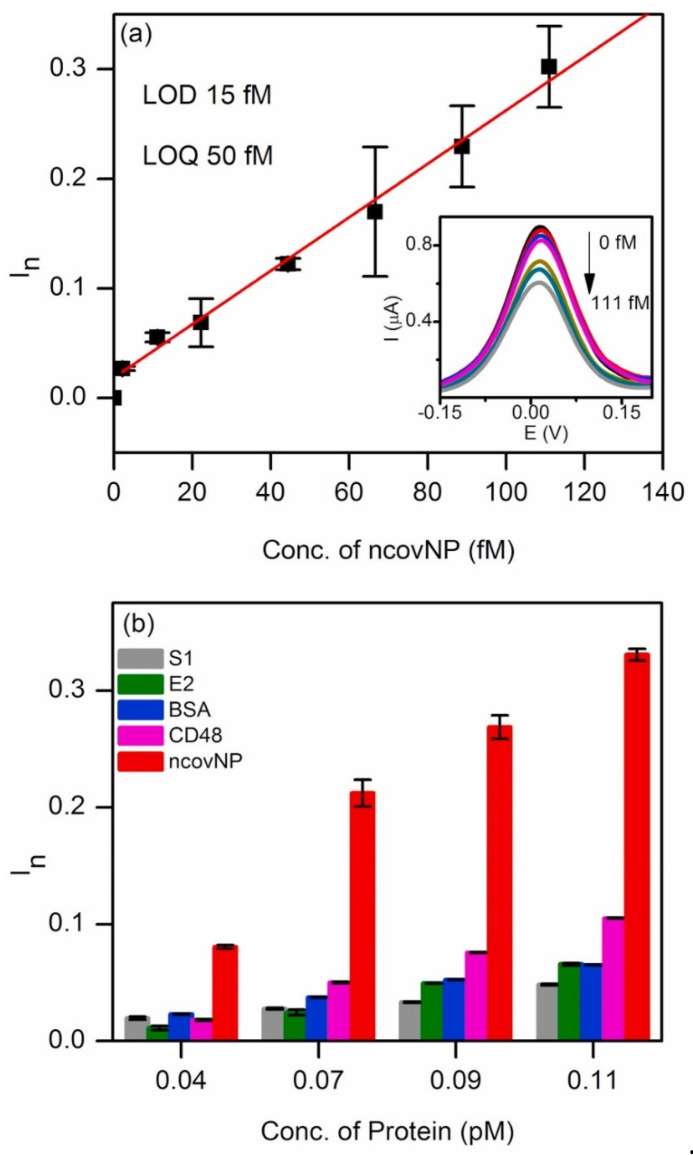
(**a**) Calibration plot of ncovNP sensor obtained at the low concentration range of ncovNP (2–111 fM) in LB. The inset shows typical DPV curves used to construct the calibration plot; (**b**) Selectivity test of ncovNP sensor showing its responses against the different proteins (S1, E2 HCV, BSA, CD48 and ncovNP) applied at concentrations (0.04, 0.07, 0.09, and 0.11 pM) in LB. ([54]).

Human immunodeficiency virus (HIV) weakens the immune system of an individual by targeting a type of white blood cell, and its early diagnosis has an important impact on the prognosis, prevention of the spread of disease in the community, treatment, and safety of blood & tissue donations. A highly selective, sensitive MIP electrochemiluminescence (MIP-ECL) sensor with Europium sulfide nanocrystals (EuSNCs) as a signal-producing compound was developed as a novel sensor for HIV-1 detection by Babamiri et al. Firstly, HIV aptamer (HIVssDNA) as a template sequence was synthesized by covalent bonding to polyacrylic acid (PAA) stabilized by EuSNCs. Then, the template was electropolymerized with *o*-PD directly on the surface of the ITO electrode. The ECL signal was endowed by hybridization reaction between oligonucleotides as detection target (HIV gene) and capture probes between assemblies of EuSNC functionalized 5-amino labeled oligonucleotides and using K_2_S_2_O_8_ as co-reactant. The developed ECL sensor demonstrated high selectivity and sensitivity for HIV gene detection due to strong ECL emission of EuSNCs [53]. The novel coronavirus, severe acute respiratory syndrome-coronavirus-2 (SARS-CoV-2), is the causative agent of the 2020 worldwide coronavirus pandemic. The nucleoprotein (NP) of the SARS-CoV-2 is an immunodominant antigen of the virus. The SARS-CoV-2 nucleoprotein (ncovNP) imprinted polymer was synthesized with poly-*m*-phenylenediamine (PmPD) as a functional monomer over p-ATP-modified Au thin-film metal electrode (Au-TFME) coupled to a portable potentiostat by Raziq et al. The analytical performance of this sensor was investigated in redox probe K_3_[Fe(CN)_6_]/K_4_[Fe(CN)_6_] by using DPV. The developed electrochemical sensor worked well towards ncovNP in the lysis buffer (LB) and achieved LOQ of 50 fM due to the presence of highly selective ncovMP antibodies, which could bind and differentiate between molecules and detect SARS-CoV-2 nucleoprotein (ncovNP). Figure 5 depicted the calibration curve and selectivity. Furthermore, the proposed sensor exhibited a pseudo-linear response vs ncovMIP for COVID-19-negative clinical samples between 0.22–333 fM. Any amount greater than 0.22 indicated COVID-19 positivity [54].

In another report for the rapid and early detection of SARS-CoV-2, an MIP-based electrochemical sensor for quantitative detection of the biomarker SARS-CoV-2 spike protein subunit S1 (ncovS1) was fabricated on Au-TFME. Selectivity of the Au-TFME-MIP sensor towards S protein subunit S1 (ncovS1) was attained by the formation of reversible covalent bonds between diol groups of highly glycosylated protein ncovS1 and boronic acid groups of 3-aminophenyl-boronic acid (APBA). With a response time of 15 min, the sensor was capable of detecting ncovS1 in both PBS buffer and nasopharyngeal swab samples, with LOD values of 15 fM and 64 fM, respectively, via square wave voltammetry (SWV) analysis using a K_3_[Fe(CN)_6_]/K_4_[Fe(CN)_6_] redox probe. Moreover, compatibility of the sensor with a portable potentiostat enabled rapid and early diagnosis of COVID-19 patients [55]. An MIP-based electrochemical sensor for determining the receptor-binding domain (RBD) of the severe acute respiratory syndrome coronavirus 2 (SARS-CoV-2-RBD) was developed by the electropolymerization of SARS-CoV-2-RBD molecules with *o*-PD over a macroporous gold screen-printed electrode (MP-Au-SPE). To determine the concentration of SARS-CoV-2-RBD in saliva, the EIS technique was used; this demonstrated that Langmuir isotherm binding was fitted satisfactorily, exhibiting an apparent dissociation constant (K_D_), maximum binding capacity (B_max_), and Gibbs free energy (ΔG) of desorption bind of 2.7 pg mL^−1^, 3.35 kΩ and -65.96 kJ mol^−1^, respectively, to detect the SARS-CoV-2-RBD. In addition, selectivity factor (α) value of >1 also indicated binding affinities of the target analyte (pg mL^−1^) in the presence of interfering antibodies (0.5 ng mL^−1^) [56]. 

### 3.4. Fertilizers

Hexachlorobenzene is used as a fungicide. Although it is effective at killing fungi, it also affects the fruit. Due to their lipophilic nature, the molecules bioaccumulate in the food chain, therefore requiring accurate and selective identification. Hence, the fabrication of the electrode was carried out using several monomers such as 1,4-diacryolyloxybenzene, 1,5-bis(2-acetylaminoacryloyloxy) pentane, and benzyl methacrylate with hexachlorobenzene. Then, a cross-linked polymer film was deposited on the surface of Quartz Crystal Microbalance (QCM) chips via spin coating. It was observed that 1,4-Diacryolyloxybenzene worked as an electron-rich host for the electron-poor hexachlorobenzene through π stacking, whereas 1,5-bis(2-acetylaminoacryloyloxy)pentane improved adhesion of the thin film to the surface of gold, thereby producing a potent MIP coating for recognition in water. This MIP-QCM was found to be very sensitive for detecting hexachlorobenzene with picomolar LOD [57]. Mazouz et al. demonstrated the fabrication of glyphosate (Gly), the most common herbicide used globally, by imprinting a thin film sensor with polypyrrole matrix (PPy) obtained at lowered oxidation potential over an Au electrode. The analytical study via surface acoustic wave (SAW, gravimetry) and electrochemical sensing (SWV) showed that the MIP-based sensor was able to selectively detect glyphosate within a linear range of 1 pM–1 nM and LOD of 1 pM. The two site binding model (K_D1_ and K_D2_) was employed to confirm the degree of interaction between MIPs and Gly molecules for gravimetry and electrochemical sensing. The values of ~ 1.4 pM and 0.67 µM for gravimetry and 2 pM and 0.57 µM for K_D1_ and K_D2_ were obtained in addition to the ΔE of −145 kJ mol^−1^ (variation in K_D1_ and K_D2_ for two sensing methods arose due to different ionic strengths of the medium). It was discussed that the low value of K_D1_ is comparable to biological counterparts owing to the complementary functionalities, size, and shape of cavities for the template. The results of the two-site binding model were compared to a novel combined model (Hill model at nanometer scale) that considered an empiric ponderation exponent for K_D1_ and K_D2_ calculation. The novel model offered a better fit and slightly different values of K_D1_ and K_D2_ [58]. Carbofuran is a broad-spectrum pesticide. The leaching of carbofuran residue in farm products and the environment is a potential threat to human beings since it disrupts endocrine activity, leading to cytotoxic and genotoxic abnormalities and reproductive disorders. Therefore, there is a demand for the effective determination of carbofuran. Li et al. employed a dual recognition element by integrating MIPs and graphene oxide adhered to AuNPs with DNA aptamers in a microfluidic chip for carbofuran determination. During analysis, the carbofuran was captured and recognized in the first identification of the chip. Then, the carbofuran was eluted off and transported to the DNA aptamer which selectively captured it and generated electric signals. Thus, this dual recognition strategy exhibited an LOD of 67 pM [59]. Chlorpyrifos (CPF) is a chlorinated organophosphate pesticide that is used extensively in agriculture due to its low cost and effectiveness against a broad spectrum of insects and pests. Even though it is very potent in controlling pests and increasing the yield of crops, it is toxic to human and animal life. It can cause several lifestyle-related and physiological disorders such as neurological or endocrine disruption, and reproductive abnormalities. Due to this, it is essential to monitor the role of CPF residues in human health. Roushani et al. demonstrated the development of a highly sensitive aptasensor MIP for CPF. Firstly, they prepared a CPF–aptamer complex with two functional monomers including *o*-PD and *o*-dihydroxybenzene (*o*-DB) followed by electropolymerization on the Au nanorod (AuNRs)-deposited GCE by applying a potential range between 0 to 1.0 V for 10 cycles (scan rate of 50 mV s^−1^) to achieve the aptamer-MIP/AuNP/GCE sensor. This electrode then was washed with methanol and nitric acid to remove CPF. At each immobilization step, CV and EIS were used to evaluate the surface features. The study reported by Roushani et al. demonstrated that under optimal working conditions, the aptamer-MIP/AuNP/GCE sensor depicted an LOD of 0.35 fM [60].

### 3.5. Explosive Materials

**Figure 6:**
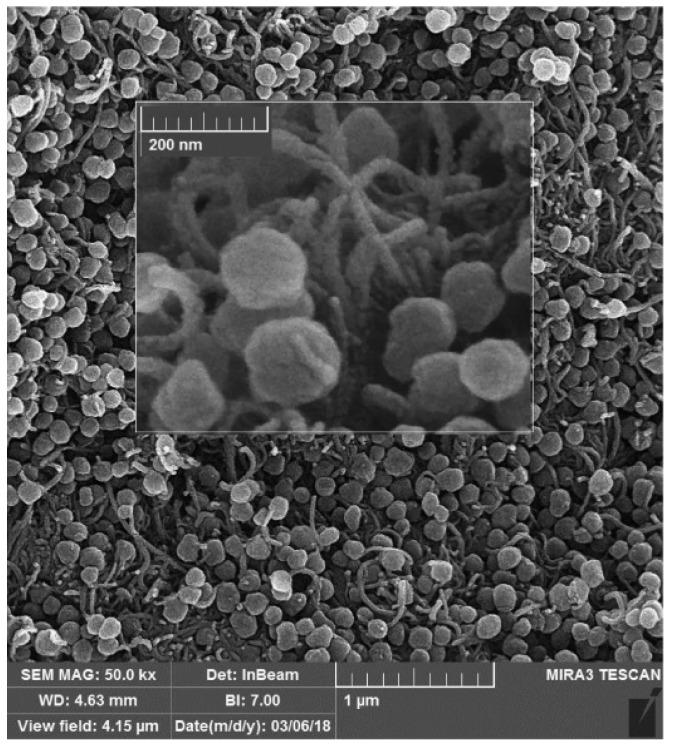
Scanning electron microscopy images of prepared nano-MIP/MWCNTs nanocomposite, cast on GC electrode ([64]).

Explosive materials belong to the class of nitroaromatic compounds; 1,3,5-trinitrotoluene (TNT) is one of the best-known explosives. Zhong Guo et al. developed an effective electrochemical sensor for the detection of TNT. Initially, a self-assembling monolayer of p-ATP was formed on the Au electrode by Au-S bonding followed by microporous-metal-organic framework (MMOF) film deposited by electropolymerization in the presence of TNT. Embedding the template in the 3D AuNPs polymer network was facilitated by π–π interaction between the electron-rich p-ATP and electron-poor TNT. It is worth noting that the LOD of this newly developed sensor reached femtomolar level, owing to the synergistic contribution of MMOF and selectivity of MIPs [61]. Fast and accurate detection of 2,4,6-Trinitrotoluene (TNT), a lethal explosive, is crucial in ensuring national security. Roushani & Shahdost-fard reported an effective sensor to detect TNT accurately, wherein an aptamer along with TNT imprinted with dopamine as a monomer via electropolymerization was immobilized on an AuNPs-fullerene (C_60_)-modified GCE. The synergistic effect of molecular imprinting and aptasensing leads to high specificity and sensitivity, providing an outstanding LOD of 3.5 aM for TNT determination [62]. Among the most widely used solid-state explosives, 3,5-trinitroperhydro-1,3-5-triazine (RDX) is more devastating than TNT. Conventional detection methods cannot be employed to detect RDX because of its extremely low saturated vapor pressure (4.85 ppt). Therefore, there is an ongoing quest to find sensors for its accurate detection. An electrochemical sensor based on MIP was developed by Alizadeh et al. for the ultra-trace level detection of RDX in aqueous samples. The electrode was fabricated with multi-walled carbon nanotubes (MWCNT) and MIP using ultrasonication with MAA and EDMA via precipitation polymerization. As shown in Figure 6, SEM studies were used to evaluate the morphology of the MIP/MWCNTs-GCE producing MIP nanospheres of 100 nm diameter with uniform distribution of MWCNTs among them. Tap water spiked with RDX was used to test the performance of the newly fabricated MIP-based electrode. It was proven by this study that MIP/MWCNTs-GCE had high selectivity with a picomolar LOD for RDX [63].

### 3.6. Antibiotics

Tetracycline (TCs), an antimicrobial broad-spectrum antibiotic, is commonly used in beekeeping to control bacterial diseases such as European and American foulbrood. For TC detection in honey, a molecularly imprinted microporous metal-organic framework (MMOF) was prepared by electropolymerization of poly-thioaniline AuNP films onto the p-ATP-modified Au electrodes. Later, LSV was used to analyze the electrochemical sensor in the presence of K_3_[Fe(CN)_6_]/K_4_[Fe(CN)_6_] as a redox probe. Under optimized conditions, the sensor depicted a linear range between 224 fM to 22.4 nM with a LOD of 0.22 fM due to the high surface area and pore volume of MMOF, the homogenous distribution of imprinted cavities, and the high electron conduction ability of AuNPs [65]. Rad et al. reported that aptamer-tetracycline complex was entrapped over a poly-dopamine-modified AuNP/GCE electrode, which developed another dual recognition impedimetric sensor for tetracycline. The resulting MIP-Aptamer exhibited higher sensing capacity than aptamer or MIP by itself and offered an LOD of 144 fM. Integration of aptamers with MIP on AuNP/GCE synergetically contributed to low LOD values [64]. Lincomycin, an antibacterial antibiotic, can be detrimental to human health, and hence monitoring the presence and concentration of Lincomycin in meat products is pivotal. A dual electrochemiluminescence (ECL) resonance energy transfer (RET)-based carbon dots (C-dots)-tagged DNA aptamer with lincomycin was electropolymerized in the presence of ***o***-aminophenol on AuNP-functionalized graphene oxide (Au-GO) nanocomposite-modified GCE. C-dots were employed as a signal indicator and in the absence of target lincomycin, the C-dots exhibited enhanced signal intensity. It was further investigated that ECL signal intensity decreased when lincomycin was competitively bound to a DNA aptamer and MIPs. The decrease in signal intensity was due to the blocked energy transfer. As a result, a dual recognition approach reported by Li et al. for lincomycin detection determined an LOD in the picomolar range [66]. Chloramphenicol (CAP), a broad-spectrum antibiotic that is effective against bacterial infections, has significant effects on human health, leading to aplastic anemia, gray syndrome, and leukemia. Hence, determining CAP residue concentration in serum and animal-derived food is vital. A dual recognition aptamer–MIP sensor on the GCE surface was fabricated for the detection of CAP. In the dual sensor, 3-aminomethyl pyridine functionalized rGO (3-ampy-rGO) was coated on the GCE surface. Later, AgNPs were immobilized on the 3-ampy-rGO/GCE surface. Consequently, this led to covalent interactions of the aptamer-CAP complex with resorcinol as functional monomer via electropolymerization on AgNPs/3-ampy-rGO/GCE surface via Ag-N bond formation. After CAP removal, the dual sensor developed by Roushani et al. manifested higher selectivity and sensitivity to CAP due to the integration of MIP with the aptamer [67]. Excessive intake of Nitrofurazone (NFZ), a broad-spectrum topical antibiotic for treating skin infections, can lead to potentially harmful effects on human health; hence, detection of residual NFZ is paramount. Thus, Lu et al. demonstrated the fabrication of NFZ imprinted polymer with bifunctional monomers comprised of nicotinamide (NA) and 2-amino-5-mercapto-1, 3, 4-thiadiazole (AMT) over a nanocomposite composed of functionalized-MWCNTs and zeolitic imidazolate frameworks (ZIF). The proposed porous sensor showed a broad detection range and LOD in the picomolar range. Such a low LOD was achieved due to the synergistic effect of enhanced recognition ability and affinity of imprinted cavities for NFZ owing to the bifunctional monomers, porous nature of ZIF, and improved electron conduction capability of MWCNTs. The standard addition method was employed for real samples (urine and water) analysis, indicating satisfactory recoveries [68]. 

### 3.7. Mycotoxins

Patulins (PAT) are mycotoxins and secondary metabolites produced by various filamentous fungi. They have been considered potential contaminants found mainly in rotting apples, apple-derived products such as juices, ciders, and other fruit juices. Many countries have set a limit for the presence of PAT in fruit juice ranging from 30 to 50 ppb. Therefore, Wei Guo et al. electropolymerized a dummy template (i.e., 2-oxindole) with *ρ*-aminothiophenol (*ρ*-ATP) over AuNPs, carbon dots (CDs), and chitosan (CS)-modified GCE. Due to the triple modification of CS, CDs, and AuNPs the sensor showed remarkable detection capability, with an LOD of 75 pM with high selectivity. For cost effectiveness, the dummy template was used. In our opinion, the usage of a dummy template also improves selectivity by reducing the non-specific binding and cleavability of the target from the polymer matrix. [69]. Fumonisins (FB1), are environmental mycotoxins produced by fusarium species grown in agricultural fields or during storage. MIP-based nanoparticles (nanoMIPs) were designed and fabricated for FB1 recognition using solid-phase synthesis, where glass beads were activated, immobilized with FB1, and polymerized via free radical polymerization in the presence of two temperature-sensitive monomers, such as N-isopropylacrylamide (NIPAM) and N-tert-butylacrylamide (TBAM). Then, the nanoMIPs were electropolymerized using a conducting polypyrrole-(zinc-porphyrin) (PPy/ZnP) film covalently bonded on a Pt electrode. On the other hand, melamine was used to synthesize non-imprinted polymer (NIP). Due to the use of NIPAM and TBAM, the FB1 was cleaved easily at an elevated temperature that offered a huge number of accessible imprinted cavities over glass beads. At the same time, low-affinity nanoMIPs and unreacted monomers were removed using low-temperature water (4 °C). Analytical signal transduction was accomplished using EIS and DPV exhibiting a sensitivity of 0.442 kΩ/pM and 0.281 μA/pM, respectively, with an imprinting factor of 6.28 with DPV. The kinetic analysis results revealed that the 1:1 Langmuir binding model was followed and appreciable selectivity was offered due to the K_D_ value of 0.2 µM and −255 kJ mol^−1^ of ΔG [70].

### 3.8. Heavy Metals

A naturally occurring heavy metal, Lead (Pb^2+^) toxicity can lead to renal, reproductive, and nervous system failure. Hence, monitoring the concentration of Pb^2+^ in environmental and biological samples is required. For selective sensing of Pb^2+^ ions, nano-structured ion imprinted polymer (IIP) with MWCNTs and carbon paste electrode (CPE) was fabricated. Precipitation polymerization technique was used for the synthesis of Pb^2+^-imprinted polymer nanoparticles by copolymerization of itaconic acid-Pb^2+^ complex and ethylene glycol dimethacrylate as a cross-linker. IIP/MWCNT-modified electrode reported by Alizadeh et al. displayed a picomolar detection of Pb^2+^ with high selectivity [71]. Cadmium II (Cd^2+^) is considered an environmental pollutant, and its increase is a worldwide concern due to its highly toxic properties causing osteoporosis, itai-itai disease, and other ailments at excessive amounts. For the detection of Cd^2+^_,_ a dual recognition aptamer-MIP sensor was fabricated by Li et al. For the synthesis, a Cd^2+^ DNA aptamer sequence was obtained from systematic evolution of ligands using the exponential enrichment (SELEX) method and polymerized with -Alanine as the functional monomer and *N*-hydroxysuccinimide as the cross-linking agent. Then, carbon quantum dots (CQDs) co-doped with sulfur and nitrogen (SN-CQD) and AuNP-modified ITO were immersed into the aptamer-MIP cocktail to obtain the intended sensor. The sensor developed by Li et al. was investigated via fluorescence demonstrating picomolar detection of Cd^2+^ [72].

### 3.9. Miscellaneous

**Figure 7:**
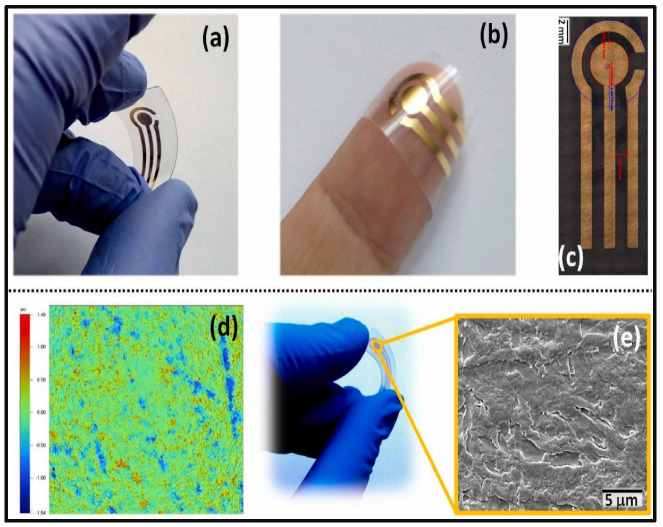
(**a**,**b**) Photographs of flexible and bendable Au electrodes on surface-roughened PEN with (**c**) dimensions of the electrodes. The size of the working electrode was 13 mm^2^, with a counter electrode of 4 mm external radius at 1 mm distance; (**d**) Typical 2-D image of the 3-D interferometric profiler analysis for an Au surface on a flexible PEN surface roughened with 12 μm abrasive paper and (**e**) SEM image of the surface of the gold working electrode (10,000× magnification). (For interpretation of the references to color in this figure legend, the reader is referred to the Web version of this article) ([78]).

In this section, we discuss the analytes which cannot be categorized specifically in any of the above-mentioned categories. Gupta et al. developed a Staphylococcal enterotoxin B (SEB) (a common contributor to food poisoning in humans)-imprinted SPR sensor. MIP and NIP films were prepared by in-situ electropolymerization of 3-aminophenylboronicacid (3-APBA) on an Au chip with and without staphylococcal enterotoxin B (SEB) molecules, respectively. SEB-MIP based SPR sensor demonstrated an LOD beyond femtomolar concentration. The kinetic parameters were assessed for the affinity of SEB with MIP indicating the K_D_ and B_max_ values of 24 fM and 71, respectively. In addition, the ΔG was found to be −77.54 kJ.mol^−1^. All these factors pointed to high rebinding affinity and spontaneous interaction between SEM and SEM-MIP. The selectivity efficiency of SEB-MIP of 100% as compared to ~23.5% for structurally related toxins substantiated the fact that SEB-MIP offered highly accessible, homogenous, and specific binding cavities [73]. Hepcidin-25 (hepcidin) is a molecule that regulates iron metabolism. Cenci and colleagues employed epitope imprinting, where the N-terminus hexapeptide of Hepcidin-25 with sequence DTHFPI was used as a template. The MIP nanoparticles (MIP-NPs)-based SPR sensor was developed using MAA/template with bisacrylamide as a functional monomer by precipitation polymerization that resulted in a 20–50 nm size of MIP-NPs for hepcidin determination. Computational modeling assisted in finding stronger interaction between the MAA/template over diethyl aminoethyl methacrylate (DEAEm)/template. The synthesized MIP-NPs reported by Cenci and colleagues were immobilized on an SPR chip that allowed the determination of Hepcidin-25 at picomolar concentrations in a short span of time. The isothermal titration calorimetry (ITC) technique thermodynamic data exhibited a K_D_ of 13 nM and a ΔG value of 51 kJ mol^−1^ corroborating the epitope strategy, high template-MIP interaction, and spontaneous interaction. Based on these parameters, 2 µg mg^−1^ of B_max_ was obtained for MIP-NPs. [74]. Urea, an essential component in biological species and the environment, is detected by an MIP-aptamer-based sensor. In this, urea was electropolymerized with dopamine as a functional monomer over gold nanoparticle and carbon nanotube (AuNPs/CNT) composite on GCE by Yarahmadi et al. Owing to the high affinity for aptamer of the target and specific binding of urea-imprinted cavities, the proposed impedimetric biosensor exhibited a high linear sensitivity with a low LOD of 900 fM [75]. Parnianchi et al. detected bilirubin (BR), an indicator of liver function in saliva, using a low-cost and non-invasive MIP. Prior to the imprinting of BR with *o*-PD via electropolymerization, MWCNTs were employed for electrode modification. The developed sensor demonstrated a high level of selectivity and a sensitivity of 1.05 µA fM^−1^ [76]. L-Tyrosine (TYR) is an amino acid, elevated concentrations of which are found in Diabetic foot ulcers (DFU). DFU can be fatal and may lead to amputation. Hence, Roy et al. prepared a templated over-oxidized imprinted PPy sensor (MIPPy) on ITO as a selective sensing system for the early detection of TYR. Moreover, the electrochemical properties of the MIPPy sensor were studied using EIS and CV methods, revealing the picomolar detection limit [77]. The compound 3-nitrotyrosine (3-NT), is a potential oxidative stress biomarker for major neurodegenerative diseases. For 3-NT detection, Martins et al. using flexible Polyethylene naphthalate (PEN) sheets developed MIP with a 3-electrode flexible and wearable point-of-care (POC) system. Firstly, the Au electrode surface was modified with thiol solution to provide a strong covalent attachment necessary for a stable self-assembled monolayer (SAM) layer as illustrated in Figure 7.

**Figure 8:**
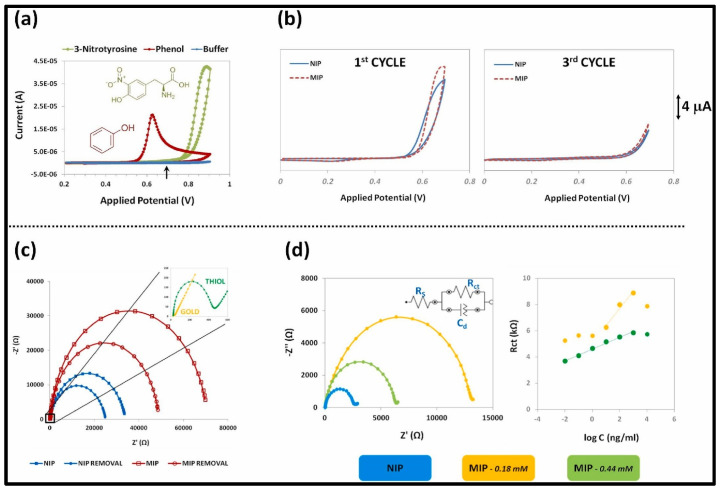
(**a**) Cyclic voltammograms of individual solutions (prepared in 100 mM of phosphate buffer solution, pH 7.2) of phenol, 3-nitrotyrosine and only phosphate buffer, at a scan rate of 20 mV/s, for one cycle; (**b**) Cyclic voltammograms for the electropolymerization of 0.30 mM phenol in 100 mM of phosphate buffer, pH 7.0, (scan rate 20 mV/s) at gold-modified electrodes with (dashed line) and without (solid line) the template molecule 3-NT for the first cycle and third cycle; (**c**) EIS curves of thiol-modified electrode NIP (blue line) and MIP (red line) before (circle symbol) and after (square symbol) template removal (inset figure with gold and thiol-modified electrodes), measured in aqueous solution containing 5 mM [Fe(CN)_6_]^3−/4−^ in 100 mM of phosphate buffer at pH 7.0; (**d**) Nyquist plots obtained for NIP and MIP materials (inset is the equivalent circuit applied) and calibration curves regarding the MIPs response after 3-NT rebinding. (For interpretation of the references to color in this figure legend, the reader is referred to the Web version of this article.) ([78]).

Later, for the fabrication of high-performance MIP films, phenol monomer was incorporated with 3-NT via electropolymerization directly on the Au surface, which allowed intermolecular interactions between 3-NT and phenol units. The preparation steps, EIS results of various steps, and a calibration curve of the 3NT via MIP are shown in Figure 8.

This flexible biosensor reported by Martins et al. demonstrated the ability to detect the picomolar amount of 3-NT with high selectivity [78]. The compound 5-hydroxyindole-3-acetic acid (5-HIAA), which is found in physiological fluids such as blood, serum, and urine, is considered a potential marker for carcinoid tumors. In addition, 5-HIAA is a metabolite of the monoamine neurotransmitter, serotonin. To detect levels of the marker, a 5-HIAA-imprinted polypyrrole (MIPPy)-based electrochemical sensor was developed by Moncer and colleagues. Electropolymerization of pyrrole with 5-HIAA via CV technique resulted in the formation of polymer film over GCE. Complementary cavities were created once the imprinted molecules have been entirely removed from the polymeric network. Under optimal conditions, the developed sensor demonstrated a high sensitivity toward the target molecule with a LOQ of 50 pM. The enhanced binding affinity between imprinted cavities and 5-HIAA was confirmed via an imprinting factor of 8.38, which was obtained from the slope ratio of MIPs and NIPs calibration curves [79].

Vitamin D3 (VD_3_) is a vital nutrient required for maintaining and building healthy bones. Its deficiency can lead to chronic health issues; hence, monitoring VD_3_ levels is essential. For selective detection of VD_3_, a highly sensitive MIP-based impedimetric sensing platform was designed by Alizadeh et al. using VD_3_ with methyl methacrylate (MMA) derivate of VD_3_ (MAVD) as a functional monomer and divinyl benzene that was subsequently incorporated into a CPE. The sensor showed high selectivity and sensitivity from 1.0 to 100.0 pM, with an LOD value of 0.22 pM [80].

Bisphenol A (BPA), a plasticizer, which can easily leach into food and water we consume through the container used to store them, is of great concern to human health. It is an endocrine-disrupting compound, which can mimic the functions of the estrogen hormone. Therefore, there is a rising demand to develop accurate and reliable sensing methods to prevent human exposure. Aptamers, short-chain single-stranded DNA or RNA oligonucleotides, can be integrated into a chemically modified electrode for the detection of BPA as shown by Ensafi et al. in a recent study. For this, the surface of GCE was modified by electrodepositing AuNPs followed by the deposition of BPA aptamer. Subsequently, electropolymerization of BPA in the presence of pyrrole and immobilization of the MIP was implemented to obtain MIP cavities (BPA@p-63/AuNP/GCE) where the embedded DNA sequence acted as the binding site for BPA. BPA@p-63/AuNP/GCE electrode modification at various stages of the experiment was monitored using SEM, EIS, and CV techniques. Following thorough investigation/studies conducted at various concentrations, Ensafi et al. determined that the attomolar concentration of BPA could be achieved [81].

Due to restrictions on the usage of BPA, manufacturers have started using other structural analogs such as bisphenol F (BPF) and bisphenol S (BPS), as shown in Figure 3. However, a similar or higher level of toxicity was observed for these analogs. Therefore, Kaya et al. described the fabrication of BPS-imprinted polymers in the presence of laboratory-synthesized N-methyloyl-L-tryrosine (MA-Tyr), an amino acid-based functional monomer in the presence of a cocktail solution including other MIPs over GCE. Analytical performance of MA-Tyr@MIP/GCE was validated by CV and DPV techniques for BPS in synthetic human serum and plastic bottled water samples. It was found that the electrochemical sensor has a higher imprinting factor (IF, MIP/NIP) for BPF (2.924) compared to BPA and bisphenol B (BPB), with BPS exhibiting LOQ of 0.762 fM in serum. The results suggested that two vital parameters, the incorporation of porogenic agent polyvinyl alcohol (PVA) and MA-Tyr, were responsible for such a high LOD. The PVA induced sufficient porosity, thus providing accessible imprinted cavities, whereas MA-Tyr enhanced the selectivity for bisphenols [82]. By comparing both methods used in the detection of bisphenols, it is noted that the use of aptamer-based MIPs offered superior LOD to attomolar levels owing to highly selective features. Melamine (MEL) triamino triazine (C_3_H_6_N_6_) is a heterocyclic organic compound with protein-like properties, and is sometimes added to milk products to increase the apparent protein content. Thus, Bakas et.al. developed a melamine imprinted thin film polymer by UV graft polymerization, which can be coated on an electrochemical device. Firstly, the coupling agent, N-dimethyl amino phenyl groups (DMA) which were formed from the electroreduction of N, N-dimethyl amino benzene diazonium tetrafluoroborate salt, was employed over an Au electrode for better adherence, stability, and improvement of the sensitivity of the MIP sensing layer of the electrochemical device. For the synthesis of the polymer, melamine was copolymerized with MAA as and EDMA in presence of benzophenone. The specificity, sensitivity, and selectivity of the film towards melamine were established by square wave voltammetry (SWV) and the surface chemical composition was confirmed using X-ray photoelectron spectroscopy (XPS) studies. The developed melamine-imprinted surface layer was found to be specific, selective, and highly sensitive towards melamine in organic solvents, especially methanol, and did not show any response for interfering compounds such as cyromazine and cyanuric acid [83].

## 4. Critical Aspects of Binding Models and Imprinting in MIPs

Factors such as types of polymerization and optimization of experimental parameters are vital for successful imprinting, which is determined via several parameters such as imprinting factor (IF), selectivity factor (α), and selectivity coefficient (k), etc. [84]. Moreover, it should be a common practice to evaluate and discuss the binding affinity models as much as possible. According to some scientists, comparison between different MIPs should not be carried out under a single set of conditions (i.e., single-point characterization) but a binding isotherm should be obtained by measuring the bound and free concentration for a fixed amount of polymer with the addition of a range of analyte concentration [85,86,87,88]. The Scatchard plot is another good way to graphically represent the kinetics of ligand binding to receptors. It can be used to determine the affinity of the receptor for its ligand and the number of binding sites from one binding site in nanomolar concentrations.

We carefully screened all the references and found that few papers discussed the binding affinities models. i.e., Langmuir isotherm and the two-site binding model. Three different properties, binding site affinity (dissociation constants), binding site selectivity (imprinting factor and selectivity factor), and binding kinetics (quantum calculation for ΔG), were discussed. Binding site heterogeneity is also discussed for specific and non-specific binding interactions between imprinted polymers and their respective templates for enhanced affinity. For this, the correct stoichiometric ratio of template/monomer, synthesis of a new monomer or choice of best-fit monomer and types of solvent were a few selected features studied for strong binding affinity. In some studies, a combination of monomers was also utilized in order to obtain strong binding affinities. The porous nature of MIPs is another essential parameter that allows diffusion (binding/unbinding) of templates into the polymer matrix in addition to the benefit of shape and size selectivity of pores toward templates. Therefore, MMOs were used in some studies exhibiting high detection limits.

It is also worth mentioning that a general view of good imprinting is to consider NIP with a minimum affinity toward the analyte. However, an interesting hypothesis was proposed by the Baggiani group, who suggested that, since the imprinting effect is created due to the template that improves the pre-existing binding features of a polymer, if an NIP exhibits binding properties toward an analyte, then the corresponding MIP will produce enhanced binding events [89]. Therefore, scientists should ponder on it and compare their results from this point of view as well.

It is well known that the magnitude of the K_D_ value relates to the concentration of ligand bound to the receptor, so the lower the K_D_ value (lower concentration), the higher the affinity of the receptor. One often-missed critical question is how protein biomarkers in the sub-nanomolar concentration range can be measured by MIP sensors which typically have 10 percent cross-reactivity in the presence of multi-fold excess of sample constituents or the problem of the molecular basis of the required affinity and the influence of cross-reactivity in real samples. It should be noted that the sub-nanomolar (pM or fM) value of K_D_ indicates superior affinity between the receptor and analyte and it is usually applicable for very high-affinity antibodies [90]. For MIP-based systems, achieving such high LODs (pM or fM or greater) cannot be easily understood. Nevertheless, the molecular basis of the affinity for measurements in the pM and fM concentration region is not yet understood. More experimental and computational models are still required to delve into this area.

## 5. Conclusions and Future Perspectives of MIPs Achieving Ultrasensitive LODs

In this review, we have comprehensively discussed diverse MIP-based electrochemical sensors and biosensors that have been effectively reported for monitoring a variety of analytes that include steroids, proteins, viruses, fertilizers, explosive materials, antibiotics, environmental contaminants, and other molecules of biological importance. Furthermore, the MIP-based sensors reported for these analytes are efficient in terms of detection and quantification in picomolar (pM) concentrations and higher. It is worth mentioning that low detection limits and higher sensitivity were achieved using aptamers, polymeric and other nanomaterials (carbon dots, quantum dots, gold, silver nanoparticles, and carbon nanotubes).

The determination of the sub-nanomolar concentration range of proteins is a daunting task. Therefore, a number of approaches including surface imprinting, porous hierarchical imprinted film, and enhanced active surface area that allows comparatively easy and selective access for the protein analyte have been proposed to overcome the existing issues. Despite several advantages, including high selectivity, variety of preparation protocols, excellent shelf life, and sustainability under extreme experimental conditions, MIPs face many hurdles in providing ultrasensitive detection limits. The issues of non-specific binding, non-uniform polymer particle sizes, swelling behavior of imprinted cavities, and tedious optimization pose difficulties. Therefore, the commercialization and POC testing devices based on MIPs are still in their nascent stages. It is worth noting that there are not many reports where ultrasensitive (i.e., ≥pM LOD) detection of the analytes was achieved. It has been shown that dual recognition approaches and the use of nanomaterials and aptamers significantly affected the detection limits and assured picomolar to attomolar detection sensitivity. Nevertheless, it is an established fact that MIP-based sensors have emerged as a versatile alternate tool to biomedical/biological antibodies and as sensor probes in immunoassays. In recent years, a shift from bulk MIP to nano MIP synthesis has evolved as a viable alternative. The nano MIP sensors are diverse in terms of selectivity, specificity, stability, and cost-effectiveness and have the advantage of long-term storage. Finally, it can be safely said that MIPs have come a long way and have emerged as a viable technique offering superior selectivity with ultrasensitive sensitivity. We are sure that scientists (MIPers) would be able to unravel a successful path to produce and offer commercialization and POC testing methods with rapid response time via MIPs.

## Figures and Tables

**Table 1:** Summary of various analytes depicting linear range and limit of detection in different MIP matrices.

Matrix	Analyte	Linear Range	LOD/LOQ *	Detection or /Electrochemcial Readout Method	% Recovery in Real Samples	Ref.
MIP/AuNPs–MWNTs/GCE	Cholesterol	0.1 pM–1 nM	0.33 pM	DPV	NR	[43]
MIP-AuNP	17-β-estradiol	3.6 fM–3.6 nM	1.09 fM/3.6 fM *	LSV	river water (95.6–103.6)	[44]
3,6-diamino-9-ethylcarbazole/MIP		1 aM–10 μM	0.36 aM	EIS	human serum (96.6–104.6)	[45]
MIP/ PSNPs	Testosterone	NR	3.5 fM	SPR	NR	[46]
MIP/GO		1 fM–1µM	0.4 fM	EIS	human serum (98.6–104.2)	[47]
NiCo_2_O_4_/rGO/MIP/ITO	Follicle stimulating hormone (FSH)	0.1 pM–1µM	0.1 pM	EIS	blood samples (90–98.79)	[48]
MAAsp/MIP/GCE	Somatostatin (SOM)	10–100 fM	0.175 fM/ 0.584 fM *	DPV	serum samples (99.32–100.25)	[49]
MIP- EG-FET	Human Serum Albumin	15 fM–150 fM	13 fM	EG-FET-Potentiostat	NR	[50]
Cd-Te-MPA QDs/MIP	Myoglobin (Myo)	50.6 fM–95 pM	7.6 fM	Fluorescence	NR	[51]
Aptamer/MIP/SiO_2_@Ag NPs	Amyloid-β oligomers (AβOs)	5–10 pg mL^−1^	1.22 pg/ml	DPV	human serum (93–107.7)	[52]
EuSNCs/ECL-MIP/ITO	Human immunodeficiency virus (HIV)	3.0 fM–0.3 nM	0.3 fM	ECL	human serum (95–102.1)	[53]
ncovNP/MIP/AuTFME	SARS-CoV-2	2.22–111 fM	15 fM/50 fM *	DPV	NR	[54]
ncovS1/MIP/AuTFME		2.0–40.0 pg mL^−1^	15 fM/ 51 fM * (PBS) 64 fM/213 fM * (NPS)	SWV	NR	[55]
MPAu/MIP/SPE		2.0–40.0 pg mL^−1^	20 fM	EIS	NR	[56]
MIP-QCM	Hexachlorobenzene	NR	1 pM	QCM	NR	[57]
PPy/MIP	Glyphosate	1 pM–1 nM	1 pM	SWV& Gravimetry (SAW)	NR	[58]
Aptamer-AuNP/MIP/GCE	Carbofuran	0.2 nM–50 nM	67 pM	DPV	Chinese cabbage (95.6), chili (110.4) lettuce (101.5) tomato (94.5), apple (92.6) banana (91.2) tangerine (89.6) watermelon (102.6)	[59]
aptamer-MIP/AuNP/GCE	Chlorpyrifos	1.0 fM–0.4 pM	0.35 fM	DPV	Apple(97.65–102.5) lettuce (98.2–103)	[60]
PATP/AuNP/MIP	1,3,5--TNT	44 nM–4.4 fM	0.044 fM	LSV	tap water (96.70–102.20), river water (100.2)	[61]
aptamer-MIP nanohybrid/AuNP@C60/GCE	2,4,6-TNT	0.01 fM–1.5 µM	3.5 aM	EIS	soil (98.50–100.50) river water (97.0–100.80)	[62]
MIP/MWCNTs-GCE	RDX	0.01–1.00 µM	20 pM/ 0.2 nM *	DPV	tap water (97.00–106.0) sea water (94.00–108.0) river water (90.00–97.50)	[63]
MMOF/MIP/AuNps	Tetracycline (TC)	224 fM- 22.4 nM	0.22 fM	LSV	spiked honey (101.84–106.1)	[64]
Aptamer/MIP/Au-GCE	Tetracycline (TET)	0.5–100 pM and 1–1000 nM	144 fM	EIS	milk (94.90–106.2)	[65]
Aptamer/ECL-MIP/Au-GO/GCE	Lincomycin	5.0 pM–1.0 nM	0.16 pM	ECL	Chicken (97.2),duck (90.2) crucian (100.1), pork (89.9), crab (103.1), beef (94.5), mutton (104.5)	[66]
AgNPs/3-ampy-RGO/MIP/GCE	Chloramphenicol (CAP)	1.0 pM–1.0 nM	0.3 pM	EIS	milk (90–103)	[67]
c-MWCNTs/MIP/ZIF	Nitrofurazone (NFZ)	0.1 pM–1.0 µM	0.067 pM	CV, DPV	Urine (99.6), water (98.7)	[68]
MIP-Au/CS-CDs/GCE	Patulin	1 pM–1 nM	75 pM	CV, DPV	Apple juice (96%–98.7%)	[69]
Nano-MIP (MIP/PPy-ZnP/Pt)	Fumonisins	1 fM–10 pM	0.03 fM 0.7 fM	EIS, DPV	Maize (96–102)	[70]
IIP/MWCNTs	Lead, Pb^2+^	0.1 pM–0.8 nM	3.8 pM	EIS, SWV	sea water (95.40–101.96), river water (97.64–102.40)	[71]
SN-CQD/Au/MIP/ITO	Cadmium, Cd^2+^	20 pM–12 nM	1.2 pM	CV,EIS	Water, soil, vegetable (82.1–113.9)	[72]
MIP	Staphylococcal enterotoxin B (SEB)	3.2–25.6 pM	0.05 fM	SPR	NR	[73]
MIP-NPs	Hepcidin-25	7.2–720 pM	5.0 PM	SPR	NR	[74]
HSA	Urea	0.005–0.1 nM, 1–500 nM	900 fM	EIS	soil (98.30–104.1), water (99.5–102.0)	[75]
OPD/MWCNT/GCE	Bilirubin (BR)	12.08 fM–91.81 fM	7.80 fM	DPV	human serum (95.23–103.80), saliva (92.85–102.21)	[76]
MIPPy/ITO	L-Tyrosine (TYR)	100 fM–1 mM	1.73 pM, 6.63 pM	CV, EIS	NR	[77]
Au/MIP	3-nitro tyrosine (3-NT)	10 pg mL^−1^–1 μg mL^−1^	24.9 pM	CV	NR	[78]
MIP/GCE	5-hydroxyindole-3-acetic acid (5-HIAA)	50 pM–50 µM	15 pM	DPV	serum (99.40–100.21), plasma (99.84–100.46), urine (98.97–101.52)	[79]
MIPNPs-CPE	Vitamin D_3_ (VD_3_)	1.0–100.0 pM	0.22 pM/ 0.73 pM *	EIS	plasma samples (94.7–104.6)	[80]
BPA@p-63aptamer/AuNP/GCE	Bisphenol A	0.5 fM–5 pM	80 aM	EIS	Fresh Milk (96.0), Milk Powder (102.0), Tap Water (94.0), Pretreated water in baby glass (96)	[81]
MA-TyrMA-Tyr@MIP/GCE	Bisphenol S	1 fM–10 fM	0.17 fM/ 0.569 fM *	CV, DPV	Serum (102.9) water (98.30–101.56)	[82]
Au-DMA/MIP/GCE	Melamine	NR	1.75 pM	SWV	Milk Sample (~95%)	[83]

* LOD (limit of detection); LOQ (limit of quantitation); PBS—phosphate buffered saline; NPS—nasopharyngeal swab; ITO—indium tin oxide; SWV—square-wave voltammetry; SAW—surface acoustic wave; EG-FET—extended-gate field-effect transistor; ECL—electrochemiluminescence; QCM—quartz crystal microbalance; SPR—surface plasmon resonance; LSV—linear sweep voltammetry; CV—cylcic voltammetry; DPV—differential pulse voltammetry; EIS—electrochemical impedance spectroscopy; NR—Not reported.

## Data Availability

Not applicable.
